# Hydroxyethyl Cellulose Promotes the Mucin Retention of Herbal Extracts Active against *Streptococcus mutans*

**DOI:** 10.3390/ma15134652

**Published:** 2022-07-01

**Authors:** Shiri Livne, Sapir Simantov, Arkadi Rahmanov, Uziel Jeffet, Nir Sterer

**Affiliations:** Department of Prosthodontics, Goldschleger School of Dental Medicine, Sackler Faculty of Medicine, Tel-Aviv University, Ramat-Aviv, Tel Aviv 6997801, Israel; dr.livne@yahoo.com (S.L.); sapirs3@mail.tau.ac.il (S.S.); phenomenar18@gmail.com (A.R.); uzieljef@gmail.com (U.J.)

**Keywords:** extracellular polysaccharide, hydroxyethyl cellulose, *Streptococcus mutans*

## Abstract

*Streptococcus mutans* is considered a major cariogenic bacterium. Most anti-cariogenic dentifrices are limited by a short exposure time. The aim of the present study was to test the hypothesis that adding a mucoadhesive agent to the formulation may increase its bioavailability and efficacy. We tested the effect of adding hydroxyethyl cellulose (HEC) to an herbal extract solution containing lavender, echinacea, sage, and mastic gum, which have been previously shown to be effective against *Streptococcus mutans*. Mucin-coated wells were treated with four test solutions: saline, herbal extracts, herbal extracts with HEC, and chlorhexidine. The wells were incubated with *Streptococcus mutans* and studied for biofilm formation (Crystal violet assay), acid production (lactate assay), acid tolerance (ATPase assay), and exopolysaccharide (EPS) production using fluorescent microscopy. The results showed that the addition of HEC to the herbal extract solution caused a significant reduction in *Streptococcus mutans* biofilm formation, lactic acid production, and EPS quantity (*p* < 0.001). These results suggest that HEC may be a beneficial added excipient to herbal extracts in an anti-cariogenic formulation.

## 1. Introduction

Dental caries is a prevalent disease among youth, affecting some 45% of the population between ages 6–19 years [1]. This disease is caused by the metabolism of sugars by the saccharolytic oral bacteria that breakdown carbohydrates and dietary sugars, which are energy source yielding acidic by-products such as lactic acid that can dissolve the hydroxyapatite crystals constructing the tooth’s hard tissues in a process known as demineralization [2], which, if left untreated, may result in cavitation, pain, dental infection, and tooth loss.

*Streptococcus mutans* is considered the main cariogenic pathogen [3] due to its enhanced abilities (i) to produce highly adhesive extracellular matrix polysaccharides in a biofilm configuration, (ii) to prolong acid production (i.e., acidogenic) facilitated by the intracellular storage of polysaccharide molecules, and (iii) to survive acidic environmental conditions (i.e., aciduric). Therefore, it is often used as the test bacterium in the evaluation of preventive treatments for caries.

Preventive dentifrices for caries are often limited in efficacy due to their short exposure time. Mucoadhesive polymers such as hydroxyethyl cellulose (HEC) have been added as excipient binders to liquid formulations in order to promote the mucosal retention of their active ingredients, thus improving their bioavailability and efficacy [4]. Other researchers have demonstrated the benefits of using mucoadhesive polymers such as HEC and HPMC as sustained-release delivery systems for antibacterial agents in a mucin-coated environment that is similar to the oral cavity, such as for eye infection treatment [5].

In a previous study, we demonstrated the antibacterial activity of four herbal extracts: lavender, echinacea, sage, and mastic gum against *Streptococcus mutans* [6]. The aim of the present study was to test the in vitro effect of adding the mucoadhesive polymer HEC to a liquid-phase herbal extract formulation on the mucoadhesive retention of the active ingredients and their effect against the cariogenic properties of *Streptococcus mutans*.

## 2. Materials and Methods

### 2.1. Bacterial Strain and Growth Conditions

*Streptococcus mutans* (ATCC 27351) was cultured in BHI media supplemented with sucrose (5% *w*/*v*) at 37 °C under anaerobic conditions.

### 2.2. Mucoadhesion Bioassay

Mucin solution was prepared by stirring and dissolving commercially available pig gastric mucin (type III, Sigma, Rehovot, Israel) in saline (1% *w*/*v*) over night at 4 °C. The mucin solution was centrifuged (6500× *g*, 30 min) and filter-sterilized using a vacuum-driven disposable filtration system (0.2 μm, StericupTM, Millipore, Burlington, MA, USA). The filtered mucin (40 μL) was placed on the bottoms of 96-well microplates (Nunc) and fixated over night at 37 °C by allowing the solvent to evaporate and the mucin coating to form a gel.

Four treatment solutions were tested: (i) saline as a negative control; (ii) a herbal extract formulation (NovaBreathTM, Tree of Life) without HEC; (iii) herbal extracts formulated with HEC (0.15% *w*/*v*); and (iv) chlorhexidine (0.2% *w*/*v*) as a positive control. Tested solutions and controls (40 μL) were placed on the bottoms of the mucin-coated wells for 2 min, and the wells were washed three times with saline to remove any non-adhered materials. Following washing, a bacterial suspension (0.4 OD, 40 μL) was placed in the bottoms of the treated wells, and BHI media (0.2 mL) supplemented with sucrose (5%) was gently added to each well. Microplates were incubated anaerobically at 37 °C for 24 h (end of Log phase) to allow for biofilm formation. Biofilms were studied for biomass, lactic acid production, ATPase activity, and extracellular polysaccharide formation, as described below.

### 2.3. Colorimetric Analysis for Biomass, Lactate and ATPase

Biofilm growth was quantified using crystal violet assay [7]. Supernatant was discarded, and 200 μL of crystal violet solution (0.1%) was added to the biofilms for 1 min. Stained biofilms were washed three times with saline to remove any unbound stain, and the stain was eluted using decolorizing solution and quantified colorimetrically. Lactate production was measured using a colorimetric lactate assay kit (abcam), and the ATPase activity in the biofilms was quantified using a colorimetric ATPase assay kit (abcam). Both kits were used as instructed in their manuals. All three colorimetric assays were performed at 600 nm using a microplate reader.

### 2.4. Extra-Cellular Polysaccharide (EPS) Production Assay

In a separate experiment, biofilms were grown under the same experimental conditions as stated above using a chamber slide well system (Lab TakTM, Nunc, Goteborg, Sweden). Following growth, the supernatant was discarded, and the wells were detached from their glass base without removing the silicone gasket. The gasket was used as a reservoir and filled with 40 μL of saline with 2 μL of a green fluorescent dye that stains both live and dead bacteria (DMAO, Promokine, Heidelberg, Germany) followed by 5 μL of Congo red solution (0.1%). The glass slabs with the stained biofilms were studied using fluorescent microscopy (×400, L3201LED, MRC, Rehovot, Israel) with an excitation wavelength of 460–470 nm and a blue LED filter with a cutoff of 500 nm. EPS production in the biofilms was quantified by analyzing digital images from each biofilm using morphometric software (ImageJ, NIH, Stapleton, NY, USA).

### 2.5. Statistical Analysis

To compare the effect of the different treatments on the various parameters, ANOVA was applied. Two-tailed tests were applied, and *p* ≤ 0.05 was considered statistically significant. Experiments were performed in six replicates (n = 6).

## 3. Results

### 3.1. Biomass, Lactate and ATPase

**Figure 1:**
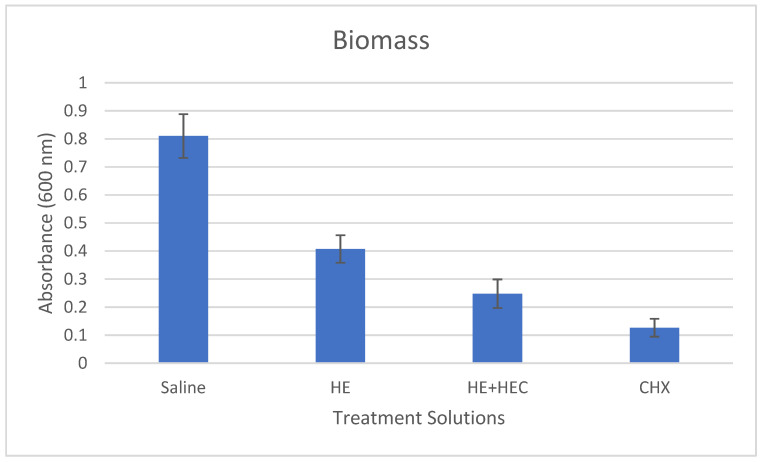
Effect of the various treatment solutions on *Streptococcus mutans* biofilm formation: saline as negative control; herbal extracts (HE); combined herbal extracts and hydroxyethyl cellulose (HE + HEC); and chlorhexidine (CHX) as positive control. Results (±standard deviation) are presented as absorbance at 600 nm.

**Figure 2:**
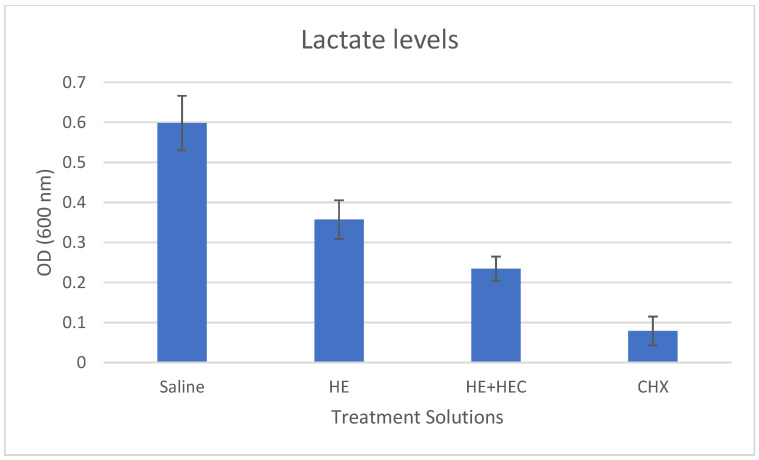
Effect of the various treatment solutions on *Streptococcus mutans* lactic acid production: saline as negative control; herbal extracts (HE); combined herbal extracts and hydroxyethyl cellulose (HE + HEC); and chlorhexidine (CHX) as positive control. Results (±standard deviation) measured using a colorimetric assay are presented as OD (600 nm) units.

**Figure 3:**
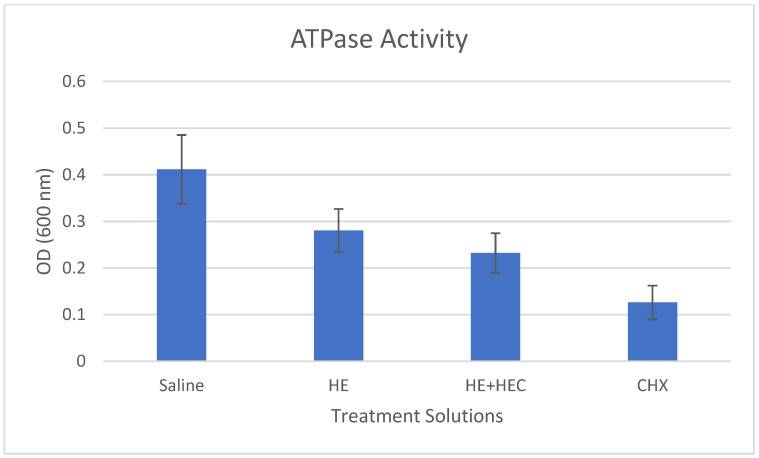
Effect of the various treatment solutions on *Streptococcus mutans* ATPase activity: saline as negative control; herbal extracts (HE); combined herbal extracts and hydroxyethyl cellulose (HE + HEC); and chlorhexidine (CHX) as positive control. Results (±standard deviation) measured using a colorimetric assay are presented as OD (600 nm) units.

The mean results of biomass formation, lactic acid production, and ATPase activity in the various treated samples are presented in Figure 1, Figure 2 and Figure 3. These results show that the addition of hydroxyethyl cellulose (HEC) significantly increased the effect of the treatment in reducing biomass formation and lactic acid production compared to herbal extracts alone (*p* < 0.001). The addition of HEC also caused an 18% reduction in the ATPase activity; however, this was not statistically significant.

### 3.2. Extra-Cellular Polysaccharide (EPS) Production Levels

**Figure 4:**
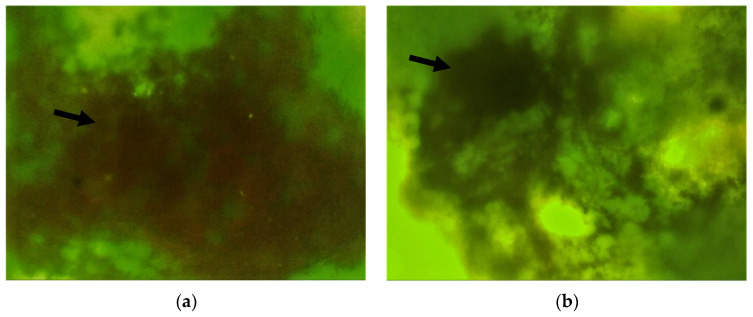

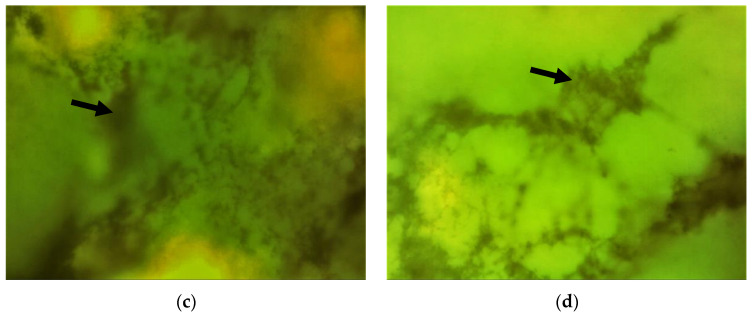
Fluorescent microscopy images showing EPS production (maroon-stained indicated by arrow) in the biofilms of the various treated samples: (**a**) saline; (**b**) herbal extracts; (**c**) combined herbal extracts and hydroxyethyl cellulose; and (**d**) chlorhexidine.

**Figure 5:**
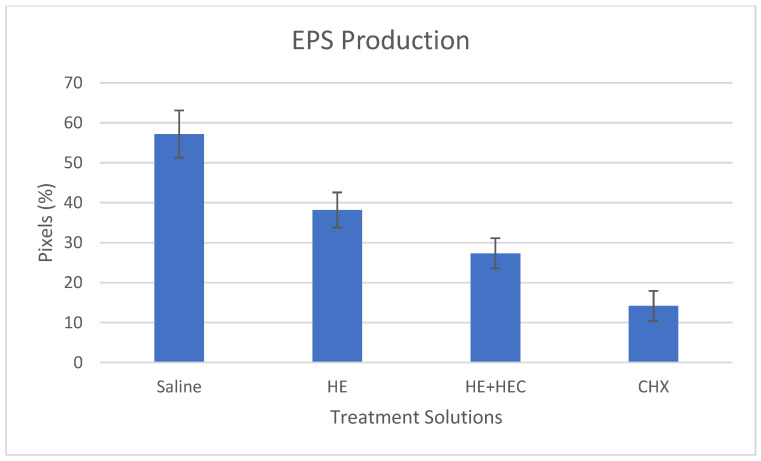
Effect of the various treatment solutions on *Streptococcus mutans* EPS production: saline as negative control; herbal extracts (HE); combined herbal extracts and hydroxyethyl cellulose (HE + HEC); and chlorhexidine (CHX) as positive control. Results (±standard deviation) are presented as percentage of maroon-stained pixels measured from digitally analyzed (Image J, NIH) fluorescent microscopy images.

The results of the fluorescent microscopy and EPS quantification are presented in Figure 4 and Figure 5. These results show a significant 30% reduction in the EPS content in the biofilm following the addition of HEC to the treatment solution compared to the non-added herbal extract solution alone (*p* < 0.001).

## 4. Discussion

We previously showed that the antibacterial effect of four herbal extracts against the cariogenic bacterium *Streptococcus mutans* [6]. The results of the current study demonstrated that adding a mucoadhesive polymer (HEC) to the herbal formulation significantly increased its mucin-retained efficacy against *Streptococcus mutans* by hindering the bacterium’s ability to form a biofilm and express its cariogenicity compared to the no-addition control. This suggests that the addition of HEC may increase the mucoadhesive binding of the herbal formulation’s active ingredients without impairing their anti-cariogenic activities.

*Streptococcus mutans* has been implicated as a major cariogenic bacterium that is responsible for the initiation and propagation [8] of carious lesions mainly through its enhanced insoluble EPS production and cariogenic biofilm formation abilities followed by its acidogenic and aciduric properties [9,10]. Therefore, antimicrobial agents play an important part in dental caries prevention and management [11]. These agents are commonly applied to the oral cavity by means of various dentifrices such as toothpastes and mouth rinses. However, due to their limited exposure time (i.e., 1–2 min), their efficacy might also be limited [12]. Hence, prolonging the exposure time is an important goal in the development of these formulations.

The use of porcine gastric mucin (PGM) as a commercially available substitute for human mucin is common in oral microbiology studies due to its considerable overall similarity to the O-linked carbohydrate sidechains of human mucins [13]. Previous studies have shown the mucoadhesive properties of HEC using PGM [14]. These mucoadhesive properties may be beneficial in prolonging the retention time and bioavailability of active ingredients used in mouthwash formulations.

The mucoadhesive properties of the HEC that was added to the liquid herbal extract in the present study combined with the increase in efficacy compared to the non-added samples suggest that some of the active ingredients in the extracts may have been bound to the mucin coating via the mucoadhesive polymer, thus preventing them from being washed and increasing their bioavailability and efficacy. This is in agreement with other researchers showing the sustained release pattern of antibacterial agents bound to HEC in an eye drop formulation [5].

Taken together, the results of the present study suggest that within the limitations of an in vitro study, adding HEC to an herbal extract formulation that is active against the cariogenic properties of *Streptococcus mutans* may increase the bioavailability and efficacy of its active ingredients via HEC’s mucoadhesive properties, thus providing a platform for the development of a caries-preventing formulation that is more efficient in reducing biofilm formation and the virulence of the cariogenic bacterium *Streptococcus mutans*. However, additional clinical investigation is warranted.

## Data Availability

Not applicable.
